# Evaluating social and spatial inequalities of large scale rapid lateral flow SARS-CoV-2 antigen testing in COVID-19 management: An observational study of Liverpool, UK (November 2020 to January 2021)

**DOI:** 10.1016/j.lanepe.2021.100107

**Published:** 2021-07

**Authors:** Mark A. Green, Marta García-Fiñana, Ben Barr, Girvan Burnside, Christopher P. Cheyne, David Hughes, Matthew Ashton, Sally Sheard, Iain E. Buchan

**Affiliations:** a Senior Lecturer in Health Geography, Department of Geography & Planning, University of Liverpool, Liverpool, UK; b Professor of Health Data Science, Department of Health Data Science, University of Liverpool, Liverpool, UK; c Professor in Applied Public Health Research, Department of Public Health and Policy, University of Liverpool, Liverpool, UK; d Senior Lecturer in Biostatistics, Department of Health Data Science, University of Liverpool, Liverpool, UK; e Research Associate, Department of Health Data Science, University of Liverpool, Liverpool, UK; f Lecturer in Health Data Science, Department of Health Data Science, University of Liverpool, Liverpool, UK; g Director of Public Health, Liverpool City Council, Liverpool, UK; h Andrew Geddes and John Rankin Professor of Modern History, Department of Public Health and Policy, University of Liverpool, Liverpool, UK; i Chair in Public Health and Clinical Informatics, Department of Public Health and Policy, University of Liverpool, Liverpool, UK

## Abstract

**Background:**

Large-scale asymptomatic testing of communities in Liverpool (UK) for SARS-CoV-2 was used as a public health tool for containing COVID-19. The aim of the study is to explore social and spatial inequalities in uptake and case-detection of rapid lateral flow SARS-CoV-2 antigen tests (LFTs) offered to people without symptoms of COVID-19.

**Methods:**

Linked pseudonymised records for asymptomatic residents in Liverpool who received a LFT for COVID-19 between 6th November 2020 to 31st January 2021 were accessed using the Combined Intelligence for Population Health Action resource. Bayesian Hierarchical Poisson Besag, York, and Mollié models were used to estimate ecological associations for uptake and positivity of testing.

**Findings:**

214 525 residents (43%) received a LFT identifying 5192 individuals as positive cases of COVID-19 (1.3% of tests were positive). Uptake was highest in November when there was military assistance. High uptake was observed again in the week preceding Christmas and was sustained into a national lockdown. Overall uptake were lower among males (e.g. 40% uptake over the whole period), Black Asian and other Minority Ethnic groups (e.g. 27% uptake for ‘Mixed’ ethnicity) and in the most deprived areas (e.g. 32% uptake in most deprived areas). These population groups were also more likely to have received positive tests for COVID-19. Models demonstrated that uptake and repeat testing were lower in areas of higher deprivation, areas located further from test sites and areas containing populations less confident in the using Internet technologies. Positive tests were spatially clustered in deprived areas.

**Interpretation:**

Large-scale voluntary asymptomatic community testing saw social, ethnic, digital and spatial inequalities in uptake. COVID-19 testing and support to isolate need to be more accessible to the vulnerable communities most impacted by the pandemic, including non-digital means of access.

**Funding:**

Department of Health and Social Care (UK) and Economic and Social Research Council.

## 1 Introduction

Severe Acute Respiratory Syndrome Coronavirus-2 (SARS-CoV-2), resulting in coronavirus disease 2019 (COVID-19), has been unprecedented in rapid global spread and impact on society. The difficulty in containing COVID-19 is in part due to asymptomatic cases making it difficult to monitor and prevent [1]. One recent study estimated that at least 50% of COVID-19 cases may have been contracted from asymptomatic individuals [2]. In response to the pandemic, the UK Government and Liverpool public health authorities piloted free rapid lateral flow SARS-CoV-2 antigen testing (LFT) for people living or working in the City of Liverpool, UK [3]. The objective was to identify cases early and break potential chains of transmission. The pilot, and subsequent extension, was intended to generate policy evidence on the performance, uptake and impacts of rapid asymptomatic testing.

**Research in context**

**Evidence before this study**

Asymptomatic transmission of SARS-CoV-2 poses a significant burden on managing the spread of COVID-19. Few studies have evaluated the impact of testing for asymptomatic COVID-19 among large populations or whole cities using empirical data. No study to our knowledge has considered if such interventions result in or exacerbate existing socioeconomic inequalities. There is a large body of evidence that demonstrates interventions that rely on human agency often widen inequalities.

**Added value of this study**

Our study provides the first substantial evidence on inequalities involved in large-scale asymptomatic rapid testing of populations for SARS-CoV-2. Data linkage to novel geospatial data reveals inequalities in the testing outcomes by deprivation, digital exclusion and accessibility to test sites.

**Implications of all the available evidence**

While testing was well received, there was a disconnect between the populations accessing testing and those experiencing harms relating to COVID-19. Provision of free and voluntary community testing requires adequate support, such as financial aid for individuals to isolate or non-digital routes for testing, to ensure inequalities are minimised.

Alt-text: Unlabelled box

The impacts of large-scale COVID-19 testing on social and spatial inequalities are unknown. Most COVID-19 testing in the UK is optional and the initial month of the Liverpool pilot encouraged all adults to “let's get tested”. Downstream interventions that rely on individual agency for engagement often exacerbate existing inequalities [4,5]. For instance, uptake of self-testing technologies for HIV is lower for Black African ethnic groups [6]. Breast and cervical cancer screening uptake, both free in the UK, are up to 10% lower in the most deprived areas than in the least deprived areas [7]. In previous pandemics such as 2009 H1N1 influenza pandemic, highly educated individuals were associated with greater likelihood of engaging in preventative, avoidant or management of disease behaviours [8]. Poor health literacy, mistrust of government, lack of free time to access services, concerns about insecure income or the inability to work from home and therefore self-isolate in the event of a positive test may all disproportionately influence disadvantaged or vulnerable groups to not get a test [9]. With deep inequalities in COVID-19 outcomes evident in the UK and globally by level of deprivation, ethnic group, and geography [10,11], testing strategies and support of people to isolate are likely to further impact on these inequalities. Concerns over whether asymptomatic testing would reach those at greatest risk were highlighted early into the pilot [12].

This study explores the social and spatial inequalities in the uptake and outcomes of large-scale rapid testing in Liverpool for people without symptoms of COVID-19.

## 2 Methods

### 2.1 Study setting

Liverpool is a post-industrial city that has high concentrated levels of deprivation and poorest health in England. Liverpool was selected by the UK Government to initially trial large-scale community testing of asymptomatic individuals for SARS-CoV-2 since it had the highest regional prevalence of COVID-19 at the time of planning. The pilot was deployed rapidly with the assistance of the British Army. The pilot was extended by request of Liverpool's public health teams (3rd December 2020), moving from a ‘mass’ (i.e., trying to test whole populations) to a SMART (Systematic Meaningful Asymptomatic Repeated Testing) approach, through targeted testing and outreach to neighbourhoods, occupations or groups at high risk (e.g., care homes) [13].

The study time period (6th November 2020 to 31st January 2021) presents an interesting context to investigate trends in testing behaviours. Liverpool had previously been placed with the most stringent regional restrictions ('Tier 3') on economic and social activities to tackle its high prevalence of COVID-19. The start of the pilot coincided with a national lockdown (5th November 2020) due to the high prevalence of COVID-19 in England. The end of the lockdown (2nd December 2020) saw lower levels of COVID-19 and Liverpool was placed into less stringent regional restrictions ('Tier 2'). Rising prevalence of COVID-19 occurred later in December across England with the emergence of the more contagious B.1.1.7 variant. While regional restrictions were made more stringent in response, populations were allowed to mix on Christmas day. A national lockdown was introduced on 6th January 2021.

### 2.2 Data

Person-level pseudonymised records were accessed using the Combined Intelligence for Population Health Action (CIPHA; www.cipha.nhs.uk) data resource. CIPHA was established in March 2020 to improve population health management for the 2·6m population of Cheshire and Merseyside (UK). It includes person-level linked anonymised records across NHS, local government, social care, administrative and public health information systems.

Our study is divided into four distinct periods reflecting the evolution of the pilot: (i) Initial ‘mass testing’ pilot period with military support (6th November to 2nd December 2020); (ii) Christmas period (3rd December to 4th January 2021), when Liverpool was one of two regions placed in Tier 2, with fewer restrictions on movement and economic activities than the rest of the country; (iii) return to national lockdown (6th January to 31st January 2021); (iv) the whole period (6th November 2020 to 31st January 2021). We selected all records that were identified as LFT for these periods. Sensitivity analyses also considered tests conducted using polymerase chain reaction (PCR) tests. Three outcome variables were defined. First, the number of people who had LFT was used to provide an indicator of uptake (i.e., the proportion of the population that had received at least one test during the time period, selecting only their first test by time period). Second, we calculated the number of people who received multiple tests (i.e., selecting their second test by time period) to identify the proportion of people who had multiple LFTs. Finally, we calculated the proportion of all LFTs by time period that were positive.

CIPHA records included for each LFT age, sex, race/ethnic group and whether an individual reported COVID-19 symptoms at their test. Missing data were low other than for ethnic group (Appendix A). Following data linkage and selecting ethnicity from repeated tests, 10·2% of individuals had missing ethnicity records. Missing ethnic group was imputed by polytomous regression using an individual's age and the ethnicity profile of their neighbourhood of residence. Addresses of individuals were matched to Lower Super Output Areas (LSOAs) to provide geographical location. LSOAs are small neighbourhood zones (~1500 people). Records were aggregated to LSOAs (n=298) to allow for analysis of geographical patterns.

To provide context for geographical patterns, we matched LSOAs to their most recently available external data on key population, social and spatial determinants of testing uptake. Official mid-year (2019) population estimates by age were used to provide denominators for uptake and account for age profiles of areas [14]. Index of Multiple Deprivation (IMD) 2019 was used to measure level of neighbourhood deprivation to identify social inequalities in uptake patterns [15]. We used deprivation score (numeric) for analytical models, and present summary statistics by Liverpool quintiles (to measure city-based inequalities) and national quintiles (to allow for wider comparisons as Liverpool is a highly deprived city). The proportion of university students in an area (numeric), using data from the 2011 Census, was included to account for targeted testing across Liverpool's universities. Whether a LSOA contained a care home or not was included (binary), using data from the Care Quality Commission (CQC), to account for targeted testing in care homes. The Internet User Classification (IUC) 2018 was selected as a proxy for confidence in using the Internet and related digital inequalities (categorical) [16]. The multidimensional measure classifies areas based on their access to Internet-related infrastructure, frequency of use, and online behaviours (e.g., ‘Digital Seniors’ or ‘e-Withdrawn’), with descriptions of each area type in Appendix D. This was due to the reliance on Internet enabled technologies for advertising the pilot, registering for tests (walk-in tests were also accepted) and receiving test results. We only consider this variable for the uptake outcome variables and not positivity, as we did not hypothesize that digital inequality would consistently affect likelihood of a positive test. Sensitivity analyses also considered an alternative measure of Internet use (see Appendix C). Finally, we estimated the street network walking distance (km) for each postcode to the nearest test site and calculated the average distance for each LSOA to account for accessibility issues that may have affected uptake (numeric). This distance was calculated at the mid time point of each of the three periods of the pilot, as the test sites that were available varied across the study period. We did not consider this variable for analysing positivity, as we did not hypothesize it would influence likelihood of a positive test. Maps of the covariates can be found in Appendix G.

### 2.3 Statistical analyses

We use a spatial regression framework to explore how our outcomes varied with the area-based factors outlined above, whilst adjusting for age, sex and ethnicity of test recipients. To account for spatial autocorrelation we used a Besag, York, and Mollié (BYM) model [17]. This Bayesian Hierarchical Poisson model accounts for the spatial nature of our data that would otherwise violate assumptions in standard regression frameworks. A separate model is fit for each outcome (modeling persons for number of tests and multiple tests, and tests for positivity) and stratified by time period (resulting in 12 models). For each spatial model, we used an indirect standardization approach to adjust for the age, sex and ethnic profile of the test recipients. First, we estimate the expected count for each outcome in each LSOA, by applying the Liverpool-wide age, sex and ethnic group specific rates for each outcome to the population estimates for each age, sex and ethnic group within each LSOA. We then included the log of these expected counts as an offset in the regression model, with the observed number of people who had a test, people who had multiple tests or number of positive tests in each LSOA as the outcome. Our area-based measures outlined above were independent variables to estimate how the relative probability of each outcome varied across these measures adjusting for age, sex and ethnicity. We also plot the predicted relative rate (observed/expected) estimated for each LSOA from our models. Models were fit using Integrated Nested Laplace Approximations (INLA) [18].

Since we only have data on people who were tested, we focus on small area patterns in testing outcomes. Due to the ecological nature of our analyses and limited ability to make inferences about individuals, we also undertook two sensitivity analyses (Appendix D) using the data on individual records for people who got a test within a binomial multi-level regression framework (individuals nested within LSOAs). First, we investigated the likelihood of an individual having had more than one test. Second, we examine the likelihood of each individual having had a positive test.

We support our analyses with additional descriptive and summary statistics to contextualize trends in testing. All analyses were conducted using R (version 3.6.2). All analytical code is available at https://github.com/markagreen/asymptomatic_testing_evaluation.

### 2.4 Role of funding source

The funders had no role in study design, data collection, data analysis, interpretation or the writing of this paper. The Department of Health and Social Care were involved in the delivery and evaluation of asymptomatic testing in Liverpool, however the decision to write and submit this paper for publication was independent of their role.

## 3 Results

Since the introduction of asymptomatic testing in Liverpool, 43% (n = 214 525) of residents aged over 5 years took 399 603 LFTs identifying 5192 likely infections or positive tests (1·3%) (Table 1; see Appendix B for descriptive statistics stratified by time period). 40% (n = 85 506; 17% of Liverpool residents) of people who got tested had multiple tests over the study period. More females (46%) than males (40%) accessed testing over the study period. Working age adults were more likely to have been tested (including 50% of residents aged 35–64), although the age group ‘15–34′ were over-represented by university students due to targeted testing during the pilot (Appendix Figure B1). There was lower test uptake among Black Asian and other Minority Ethnic (BAME) groups, especially among ‘Mixed’ (27%) and ‘Other’ (28%) ethnic groups. The percentage of positive tests was higher among ‘Black’ (2%) and ‘Other’ (3%) ethnic groups. Inequalities were observed by neighbourhood deprivation, with residents of the most deprived areas having both lower uptake (32% for most deprived vs 53% least deprived Liverpool quintiles) and a higher percentage of tests that were positive (1·74% for most deprived vs 1·04% least deprived Liverpool quintiles).

**Table 1:** Summary statistics of the three outcome measures for the whole period of analysis (6th November 2020 to 31st January 2021). Note: Ethnicity estimates are following imputation. Denominators for percentages: (i) Uptake is 2019 mid-year population estimate, (ii) Multiple tests is number of people tested, (iii) Positivity uses total number of tests.

		Uptake (persons)		Multiple tests (persons)		Positivity (tests)	
Measure		Frequency	Percentage	Frequency	Percentage	Frequency	Percentage
Total		214,525	43·1	85,506	39·9	5192	1.3
Sex	Female	114,517	45·9	47,739	41·7	2604	1.18
	Male	100,008	40·2	37,767	37·8	2588	1.44
Age band	6–14	19,491	23·6	8265	42·4	297	0.92
	15–34	78,418	46·5	29,977	38·2	2309	1.65
	35–69	96,721	49·5	40,101	41·5	2369	1.23
	70+	19,895	38·5	7163	36·0	217	0.63
Ethnic group	Asian	7279	37·5	2299	31·6	160	1.37
	Black	4899	39·8	1641	33·5	157	1.96
	Mixed	3216	27·4	1272	39·6	70	1.23
	Other	2279	27·5	672	29·5	112	3.28
	White	196,852	47·5	79,622	40·4	4693	1.27
Deprivation: Liverpool quintiles	Least Deprived	51,957	53·0	23,241	44·7	1101	1.04
	Quintile 2	51,625	49·1	21,427	41·5	1053	1.07
	Quintile 3	44,248	47·0	16,974	38·4	1089	1.37
	Quintile 4	34,679	34·5	12,774	36·8	996	1.62
	Most Deprived	32,016	31·9	11,090	34·6	953	1.74
Deprivation: England quintiles	Least Deprived	3942	58·0	1975	50·1	57	0.71
	Quintile 2	27,359	56·6	12,521	45·8	601	1.07
	Quintile 3	25,832	48·6	10,910	42·2	569	1.11
	Quintile 4	38,560	47·9	15,928	41·3	760	1.03
	Most Deprived	118,832	38·4	44,172	37·2	3205	1.52

**Fig. 1:**
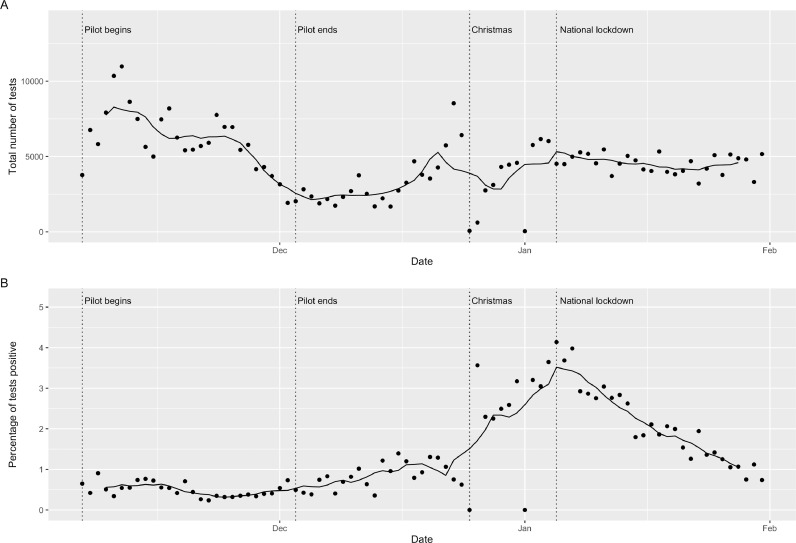
Trends in the number of lateral flow tests per day (top) and the percentage of lateral flow tests that were positive (bottom). Note: Points are daily values, line is the 7-day average.

Trends in the number of tests over time (Fig. 1) reflect initial high uptake during the initial push, declining following planned withdrawal of military assistance shortly after Liverpool's move into less stringent (Tier 2) local restrictions (announced 26th November 2020, enacted 2nd December 2020). Uptake remained initially low in December, before a sharp increase in the week before Christmas. High demand was sustained after Christmas and into the national lockdown.

Trends in the positivity rate for LFT remained consistently low (<1·5%) up to Christmas (Fig. 1). Post-Christmas there was a rapid increase in the percentage of LFTs that were positive, with a doubling of the positivity rate. Symptomatic or pauci-symptomatic individuals may have also been accessing asymptomatic testing services during this period due to easier access, quicker turnaround times for test results and habitual changes to testing behavior, including repeated testing. We examined this hypothesis through exploring trends in the percentage of individuals accessing LFTs who reported that they had symptoms at their test. A small increase in trends was observed after Christmas (Appendix Figure B2), although overall prevalence remained low (n= 1515 or 0·38% of all LFTs). Positivity rates declined following the national lockdown. Patterns for adjudication of LFT test with follow-up PCR test are presented in Appendix F. The results suggest moderate follow-up for PCR adjudication (especially during the initial pilot), although high agreement where a PCR test was completed.

**Fig. 2:**
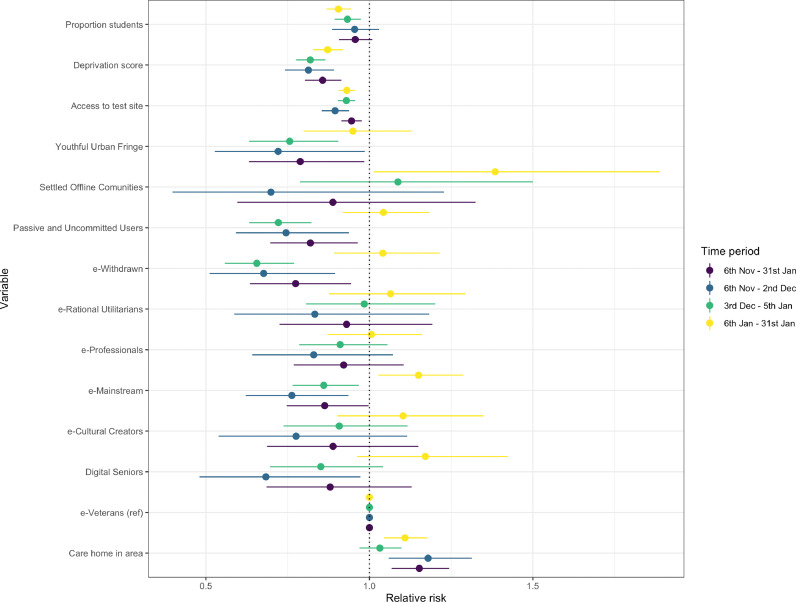
Estimated relative risks (mean and 95% credible intervals) for the associations between independent variables and uptake of tests by time period model. Note: Transparent values represent estimates where credible intervals contain 1.

Fig. 2 presents the results from the Bayesian Hierarchical Poisson model exploring the neighbourhood determinants of overall uptake patterns (see Appendix C for full models). Deprivation was negatively related to uptake, suggesting that increasing levels of deprivation were associated with lower uptake. For example, a one standard deviation increase in deprivation score (equivalent of going from Liverpool's third quintile to most deprived quintile) was associated to 14% fewer tests over the whole period (Relative Risk (RR) = 0·86, 95% Credible Intervals (CIs) = 0·80–0·91). The association was found for each period suggesting the importance of social inequalities in uptake. Distance from home to test site was also important, being negatively associated to uptake suggesting that uptake was lower among those living further from test sites (e.g. whole period RR = 0·95, 95% CIs = 0·91–0·98). Estimating the unstandardized effect size (standardised coefficient / standard deviation) to aid interpretation suggests that each 1 km increase in distance to nearest test site was associated with 11% fewer tests. Estimated effect size was largest during the pilot (‘mass testing’) period where there were more test sites. There was a negative association between the proportion of students in an area and uptake, with effect sizes largest for the two periods post-pilot reflecting that student populations were encouraged to return home in early December (e.g. 6th Jan – 31st Jan RR = 0·91, 95% CIs = 0·87–0·94). Areas that contained a care home were positively associated with uptake, suggesting that testing was higher in areas with a care home present. For example over the whole period, areas with care homes had 15% more tests (RR = 1·15, 95% CIs = 1·07–1·24).

We found the Internet-related characteristics of areas were associated with uptake, suggesting that digital exclusion was a legitimate concern. Populations less confident with using Internet technologies, as measured by the Internet User Classification, showed lower uptake. For example, areas classified as ‘e-Withdrawn’ (described as least engaged with the Internet) had 23% (RR = 0·77, 95% CIs = 0·63–0·94) lower uptake over the whole period than ‘e-Veterans’ (the group hypothesised to have the most confidence with using Internet technologies). Results were inconsistent when using an alternative measure of Internet use (Appendix D).

**Fig. 3:**
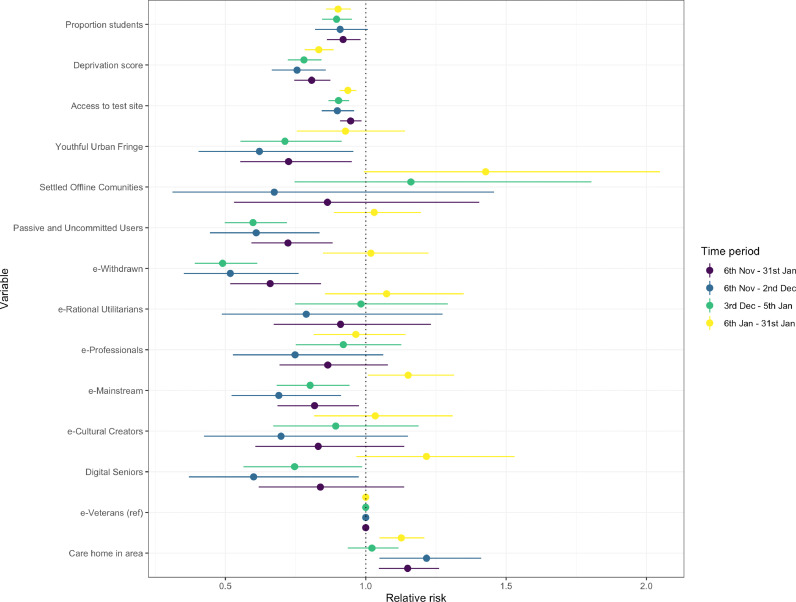
Estimated relative risks (mean and 95% credible intervals) for the associations between independent variables and multiple tests by time period model. Note: Transparent values represent estimates where credible intervals contain 1.

Analysis for individuals who had multiple LFTs showed similar results to those described above for overall uptake (Fig. 3, Appendix C).

**Fig. 4:**
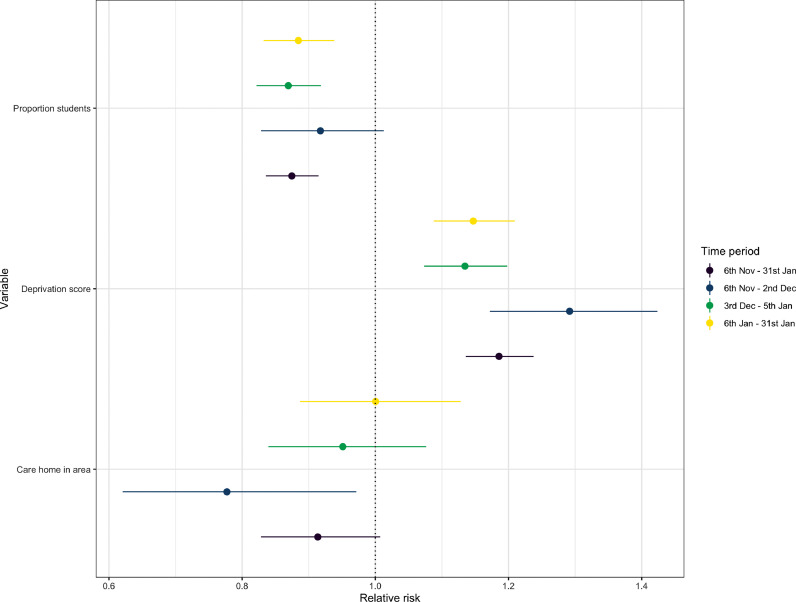
Estimated relative risks (mean and 95% credible intervals) for the associations between independent variables and positivity by time period model. Note: Transparent values represent estimates where credible intervals contain 1.

Fig. 4 presents the model results for positive tests. There was large uncertainty in associations for location of a care home in an area. Deprivation score was positively associated with positivity at each time period, suggesting that areas that were more deprived had higher proportion of positive tests. For each one standard deviation increase in deprivation score, there was an increase in positive tests by 19% (RR = 1·19, 95% CIs = 1·14–1·24). The proportion of students in an area was negatively associated to positivity for most time periods, with associations uncertain during the initial pilot period. The result suggest that a one standard deviation increase in the proportion of students in an area was associated with 13% fewer positive tests over the whole period (RR = 0·87, 95% CIs = 0·84–0·91). Estimating the unstandardized effect size here to aid interpretation would suggest that a one unit increase in the proportion of students (equivalent to comparing an area where all residents are students to those with none) would see 53% fewer positive tests.

Sensitivity analyses investigating the likelihood of having multiple tests or a positive test at the individual level revealed largely similar associations for the contextual factors described previously (Appendix E). Analyses revealed inequalities by demographic characteristics. Age was negatively associated with the likelihood of a positive test, suggesting that asymptomatic older adults were less likely to have tested positive for COVID-19. Males, compared to females, were more likely to have a positive test and less likely to have had multiple tests. Finally, the ‘Other’ ethnic group were more likely to have had a positive test, with all BAME groups less likely to have had multiple tests. Similar social and spatial inequalities were observed for whether individuals with positive LFTs also received a follow-up PCR (Appendix F).

**Fig. 5:**
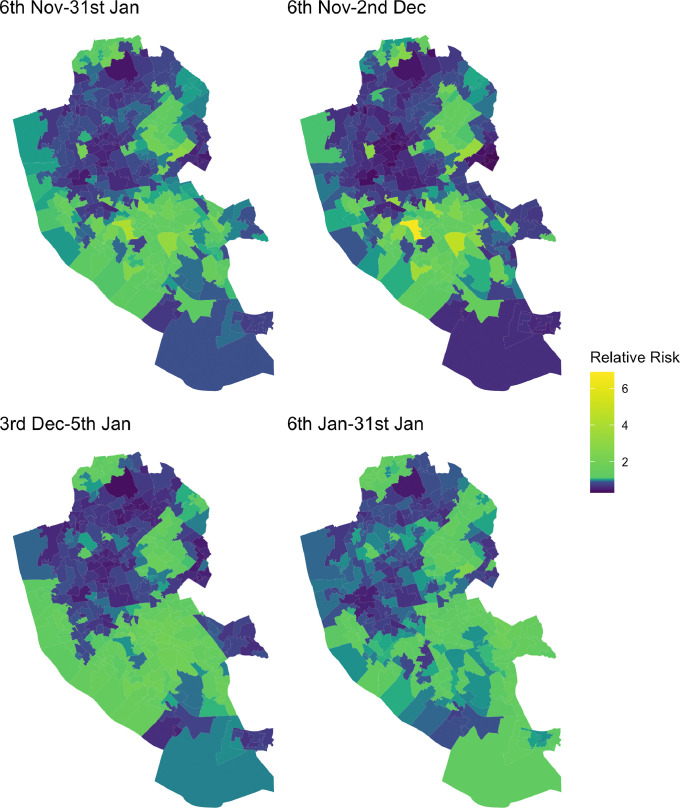
Relative uptake (observed count / expected count) for overall lateral flow test uptake for lower layer super output areas. Note: red values are relative risks <1, blue colours are >1.

**Fig. 6:**
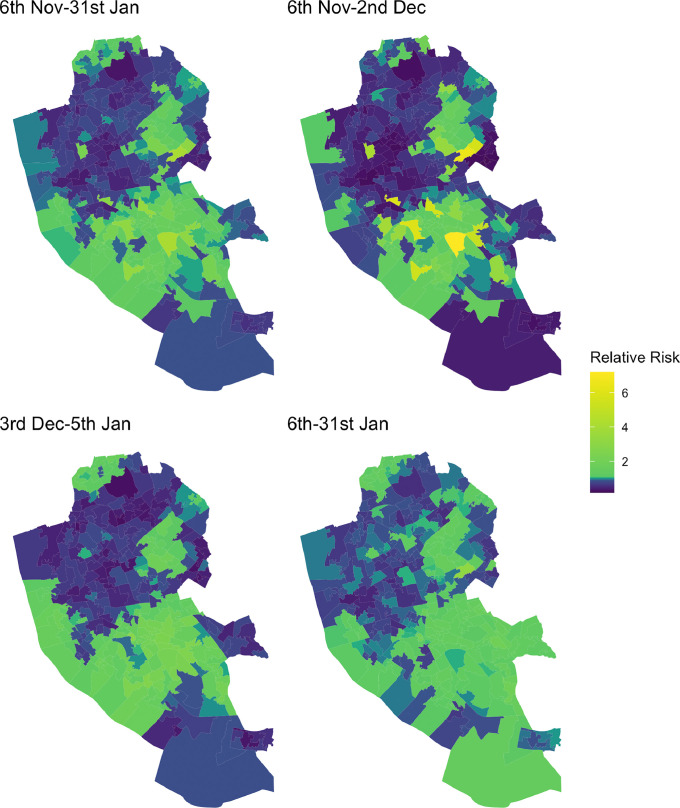
Relative uptake (observed count / expected count) for multiple lateral flow tests for lower super output areas. Note: red values are relative risks <1, blue colours are >1.

**Fig. 7:**
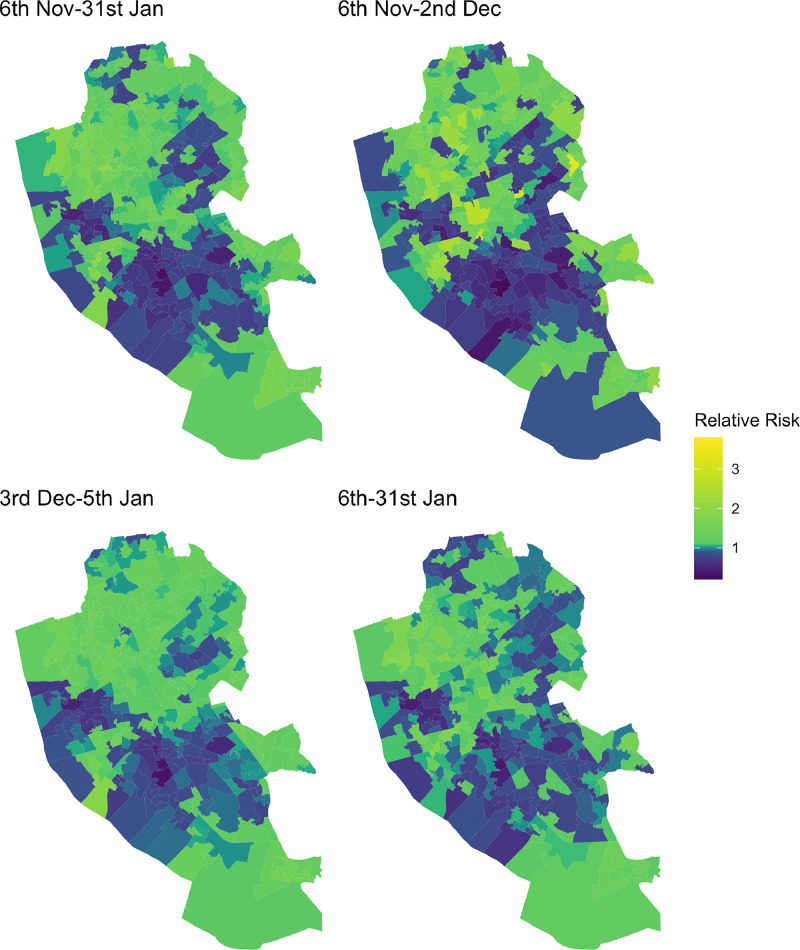
Relative rates (observed count / expected count) for positive lateral flow tests for lower super output areas. Note: red values are relative risks >1, blue colours are <1.

Fig. 5, Fig. 6 and 7 plot the geographical patterns of the outcome variables estimated from our analytical models. There were distinct geographical inequalities in uptake, with clustering of low uptake in densely populated deprived communities (Figure 5 and 6). Geographical patterns were less distinct during the national lockdown, especially for multiple tests. The geographical patterns for uptake contrasted to those for positive tests (Fig. 7), which were inversely clustered with higher positivity in deprived areas suggesting spatial inequalities were important in explaining the spread of asymptomatic COVID-19 cases. Uptake and positivity were negatively associated (with a correlation r = -0·54 for the whole study period), suggesting that areas with lower LFT uptake also had more positive tests. The patterns suggest a disconnect between the populations coming forward for testing and those at greatest risk of being infected.

## 4 Discussion

Our study provides the first substantial evidence on inequalities involved in large-scale asymptomatic rapid testing of populations for SARS-CoV-2. We find that the provision of free asymptomatic testing saw 43% (n = 214 525) of residents aged over 5 years in Liverpool receiving tests between 6th November 2020 and 31st January 2021. 1·3% of tests were positive, identifying 5192 individuals who did not know they had the virus who were notified of the need to self-isolate, potentially breaking chains of transmission. Supply and demand for asymptomatic testing was highest during the initial ‘mass testing’ period with military assistance but rose again as SMART testing was introduced with a smaller number of testing centres. Demand was particularly high in the pre-Christmas period, and sustained unexpectedly through lockdown as the advertising message shifted to testing front-line workers. We found evidence of inequalities in uptake and repeat testing, with lower uptake among deprived populations, BAME populations, areas with poor access to test sites and areas classified with high digital exclusion. Spatial inequalities were key in explaining positivity rates, with some evidence of higher positivity among deprived populations and those with low student populations.

There are strengths and weaknesses to our study. We use timely data covering all tests within Liverpool to promptly evaluate a key COVID-19 policy area with little prior evidence. Data were linked to novel geospatial information to contextualize patterns in uptake. Whilst the geospatial data were valuable, there were some discrepancies in data coverage and timing. Although neighbourhood characteristics tend to occur on longer-term trends rather than annual fluctuations [19], our analysis highlights the difficulty in the need for timely socioeconomic data for making informed decisions. Our models are cross-sectional and association-based, limiting any causal interpretation. Observations are area based and thus susceptible to ecologic fallacy, which we have attempted to mitigate in our interpretations (also see Appendix E). Analyses are undertaken for small statistical zones that may not reflect actual neighbourhoods, and their defined shapes and sizes may influence the results.

Our study shows that provision of free and voluntary asymptomatic community testing is affected by substantial social and spatial inequalities, typical of the ‘inverse care’ law but with a distinctive digital exclusion factor consistent with the digitally intensive means of accessing testing (participants are usually registered via smartphone and receive results by text message or email, with work-arounds for those without mobile phones). We found large relative inequalities by level of deprivation in uptake, repeat testing and positivity rates. Although uptake was lowest in the most deprived areas, we find that it was higher than the 4% figure shared by others and note no single LSOA had such low uptake [12]. We further identify inequalities by ethnicity and geographical location. The experiences described in our study follow a large body of evidence demonstrating how voluntary or downstream interventions that rely on individual agency often widen the inequalities they seek to tackle [4,5]. These issues are paramount given that the groups we describe as having lower uptake are the groups hit hardest by COVID-19 prevalence and related health and social outcomes [10,11]. Our results suggest that those populations which have lowest uptake tend to be those who likely need it the most.

Over one year into COVID-19 and societies are still learning how to manage this pandemic. Asymptomatic transmission is a major risk to manage [2], but there is little evidence on how to do so effectively and equitably with rapid tests of infectiousness such as LFTs. Our study adds critical and timely evidence. With national expansions planned for the UK and USA, successful management will need to proactively account for the inequalities we describe. Digital exclusion was an important barrier for uptake, and our results follow emerging evidence on how digital technologies have significant direct and indirect impacts on health [20]. Digital inclusion will therefore be key to any design of interventions, through engaging with populations less confident in Internet technologies and offering non-digital routes for testing embedded in deprived communities. As digital exclusion is often greater among deprived and vulnerable communities [16,20], interventions aimed at tackling digital exclusion may narrow inequalities. Improved communication and messaging through non-digital methods may help to alleviate concerns and encourage testing as well [9]. Accessibility was also a key factor in explaining LFT uptake demonstrating that test sites will need to be geographically accessible, convenient and account for a lack of private transport which is often more common among deprived populations [21]. Home testing may also help to minimize these issues, although there is a lack of evidence on how effective testing outside of official sites might be. Finally, we demonstrated that deprivation was an important issue in test uptake and case-detection. Emerging evidence suggests that individuals from low income backgrounds may avoid testing, not engage with contact tracing or not isolate if it meant not being able to work [11,13,22]. Greater financial support for individuals isolating may be effective here, especially for populations unable to work from home. However, testing alone may not be sufficient to support the range of issues facing deprived communities that place them at higher risk of the harms relating to COVID-19.

Avoiding inequalities in COVID-19 related outcomes is possible through carefully designed interventions, especially when combined into a comprehensive set of interventions. However, the example of asymptomatic testing in Liverpool suggests that current approaches to manage the COVID-19 pandemic may unintentionally widen inequalities through less engagement among those communities who have experienced the largest social and health-related harms of the pandemic. Learning how to effectively minimize inequalities in testing behaviours, including the mechanisms and barriers underpinning the relationships we identify, is critical if we are going to be able to effectively manage COVID-19 and future pandemics.

## 5 Contributors

MAG, IB, MGF and BB designed the study. MA, SS and IB were involved in the design, roll-out and delivery of asymptomatic testing in Liverpool. GB, DH and CC set up data systems and supported data cleaning. MAG and BB further cleaned data for analysis. MAG undertook analyses under the supervision of MGF and BB. MAG, IB, BB, MGF, GB, DH and CC provided interpretation for data. All authors contributed to writing the manuscript and agreed to submit the manuscript for publication.

## Funding

This report is independent research funded by the Department of Health and Social Care. This work was supported by the Economic and Social Research Council [grant number ES/L011840/1]. IB is supported by the National Institute for Health Research as Senior Investigator. BB was supported by the NIHR Applied Research Collaboration North West Coast. The NIHR had no role in the study design, data collection and analysis, decision to publish or preparation of the article. This report is independent research arising from research supported by the NIHR. The views expressed in this publication are those of the author(s) and not necessarily those of the NHS, NIHR or the Department of Health and Social Care.

## Ethical approval

The University of Liverpool has provided secondary data analysis as part of a national service evaluation with data collected by Department of Health and Social Care (Sponsor) for quality assurance of Innova lateral flow tests in a public health service intervention. There was no research commissioned by Department of Health and Social Care on this aspect of the Liverpool pilot of asymptomatic, community testing. As such, research ethics approval was not sought by the Department of Health and Social Care. Some aspects of the evaluation requiring fieldwork and primary data collection by the University of Liverpool were subject to ethical approval, which was confirmed prior to the commencement of activities by the University of Liverpool's Research Ethics Committee. The provision of secondary data analysis and interpretation did not require further ethical approval. Cheshire & Merseyside Health & Care Partnership Combined Intelligence for Population Health Action (CIPHA) Data Access Committee approved access to the data and analysis contained in the study. MAST/SMART was defined as ‘an emergency public health intervention during an extraordinary event’ which were subject to the legal and ethical provisions of a health protection activity and COVID-19 specifically. The secondary analysis of data provided in a health protection activity is not classified as research, and so does not require research ethics committee review (see http://www.hra-decisiontools.org.uk/research/docs/DefiningResearchTable_Oct2017–1.pdf).

## Data sharing

Data are accessible via CIPHA. Requests can be made to the Data Access Committee for extracts of the larger-scale data which cannot be released openly due to information governance requirements. All R code is accessible here https://github.com/markagreen/asymptomatic_testing_evaluation.

## Declaration of interests

IB and SS report grants from Department of Health and Social Care during the conduct of the study. IB declares grants from NIHR, personal fees and other from AstraZeneca, outside the submitted work. SS research is supported by a grant from Wellcome Trust. No other interests to declare.

## Appendix Supplementary materials

Image, application 1
