# Supplementary material for: Evaluating social and spatial inequalities of large scale rapid lateral flow SARS-CoV-2 antigen testing in COVID-19 management: An observational study of Liverpool, UK (November 2020 to January 2021)
Source: Lancet Reg Health Eur. 2021 May 12;6:100107. doi: 10.1016/j.lanepe.2021.100107 (PMC8114854; doi:10.1016/j.lanepe.2021.100107)
Supplement: Supplementary file 1 [file mmc1.pdf]

Supplementary Appendices

The document presents additional data and analyses to support the results presented in the manuscript entitled '*Evaluating social and spatial inequalities of large scale rapid lateral flow SARS-CoV-2 antigen testing in COVID-19 management: An observational study of Liverpool, UK (November 2020 to January 2021)*'.

Appendix A: Missing data

From the CIPHA database containing 970 814 lateral flow tests between 6th November 2020 and 31st January 2021, we identified 406 327 lateral flow tests for residents in Liverpool (5922 tests or 0·006% had missing address data). Investigating patterns in missing data for our analytical dataset (i.e., the 406 327 lateral flow tests) revealed no missing data for sex. All tests contained information about age, however we treated all tests with age less than or equal to 5 years as missing (1·7%) since there was no asymptomatic tests being delivered to these ages. Dropping these records from the data resulted in a sample size of 399 603. The high level of data completion was captured via users completing electronic forms on site prior to their test (recommended on their mobile phones), with additional information gathered from data linkage to NHS data systems. Where records for individuals had different recorded ages (i.e. individuals who had birthdays over the study period), we selected the earliest test. Tests with void or insufficient results (n=1625, 0·4%) were excluded from analyses.

Ethnicity had lower completed coverage with 10·2% (n = 41 545) tests with missing data post-data linkage, with individuals selecting 'prefer not to say' when registering at a test centre. We used imputation by polytomous regression to impute ethnic groups for persons with missing data using Multivariate Imputation by Chained Equations (R packaged 'mice'). The method is commonly used for imputing missing data. The approach assumes that records were missing at random and this assumption may not hold with our data. Predicted ethnic group (White, Black, Asian, Mixed or Other) was estimated based on an individual's age, sex and the composition of their neighbourhood (lower super output area). Area level predictors included the proportion of residents by each ethnic group, deprivation score, the proportion of students, and the number of care home beds per population.

Table A1: Frequency of missing data by test records for Liverpool.

Variable	Frequency	Percentage
Age	6742	1·7%
Sex	0	0%
Ethnicity	41545	10·2%
Test result*	1625	0·4%

* Refers to tests with void or insufficient results

Appendix B: Descriptive statistics

Detailed descriptive statistics for each outcome measure by time period of analysis are presented in Table B1-B3.

Table B1: Summary statistics for individuals who received at least one lateral flow antigen test by population characteristics. Denominator for percentages is 2019 ONS mid-year population estimate for each group other than ethnicity which uses 2011 Census population.

Measure		6 Nov 2020 - 31 Jan 2021		6 Nov - 2 Dec 2020		3 Dec 2020 - 5 Jan 2021		6 Jan - 31 Jan 2021	
		Frequency	Percentage	Frequency	Percentage	Frequency	Percentage	Frequency	Percentage
Total persons		214525	43·1	117549	23·6	80981	16·3	75371	15·1
Sex	Female	114517	45·9	63991	25·7	43929	17·6	41253	16·5
	Male	100008	40·2	53558	21·5	37052	14·9	34118	13·7
Age band	6-14	19491	23·6	14905	18·1	4350	5·3	3029	3·7
	15-34	78418	46·5	35803	21·2	32597	19·3	28366	16·8
	35-69	96721	49·5	52433	26·8	37776	19·3	39461	20·2
	70+	19895	38·5	14408	27·8	6258	12·1	4515	8·7
Ethnic group	Asian	7279	37·5	4026	20·7	2221	11·4	2196	11·3
	Black	4899	39·8	2437	19·8	1533	12·5	1819	14·8
	Mixed	3216	27·4	1702	14·5	1225	10·4	1072	9·1
	Other	2279	27·5	1078	13·0	752	9·1	787	9·5
	White	196852	47·5	108306	26·1	75250	18·1	69497	16·8
Deprivation: Liverpool quintiles	Least Deprived	51957	53·0	29949	30·5	22866	23·3	16752	17·1
	Quintile 2	51625	49·1	28372	27·0	20587	19·6	17309	16·5
	Quintile 3	44248	47·0	25112	26·7	15170	16·1	15241	16·2
	Quintile 4	34679	34·5	18150	18·1	11611	11·6	13296	13·2
	Most Deprived	32016	31·9	15966	15·9	10747	10·7	12773	12·7
Deprivation: England quintiles	Least Deprived	3942	58·0	2583	38·0	1593	23·4	964	14·2
	Quintile 2	27359	56·6	16316	33·7	12184	25·2	8540	17·7
	Quintile 3	25832	48·6	13603	25·6	11492	21·6	9105	17·1
	Quintile 4	38560	47·9	21423	26·6	15199	18·9	12793	15·9
	Most Deprived	118832	38·4	63624	20·6	40513	13·1	43969	14·2

Table B2: Summary statistics for individuals who received multiple tests by population characteristics. Denominator for percentages are number of people who were tested in each group (i.e., frequency values in Table B1).

Measure		6 Nov 2020 - 31 Jan 2021		6 Nov - 2 Dec 2020		3 Dec 2020 - 5 Jan 2021		6 Jan - 31 Jan 2021	
		Frequency	Percentage	Frequency	Percentage	Frequency	Percentage	Frequency	Percentage
Total persons		85506	39·9	36817	31·3	20833	25·7	22520	29·9
Sex	Female	47739	41·7	20527	32·1	11537	26·3	13140	31·9
	Male	37767	37·8	16290	30·4	9296	25·1	9380	27·5
Age band	6-14	8265	42·4	6311	42·3	774	17·8	752	24·8
	15-34	29977	38·2	10258	28·7	8689	26·7	8397	29·6
	35-69	40101	41·5	15912	30·3	10027	26·5	12432	31·5
	70+	7163	36·0	4336	30·1	1343	21·5	939	20·8
Ethnic group	Asian	2299	31·6	972	24·1	454	20·4	653	29·7
	Black	1641	33·5	611	25·1	289	18·9	536	29·5
	Mixed	1272	39·6	524	30·8	292	23·8	321	29·9
	Other	672	29·5	238	22·1	144	19·1	194	24·7
	White	79622	40·4	34472	31·8	19654	26·1	20816	30·0
Deprivation: Liverpool quintiles	Least Deprived	23241	44·7	10729	35·8	6679	29·2	5328	31·8
	Quintile 2	21427	41·5	9579	33·8	5593	27·2	5303	30·6
	Quintile 3	16974	38·4	7317	29·1	3756	24·8	4557	29·9
	Quintile 4	12774	36·8	5183	28·6	2607	22·5	3782	28·4
	Most Deprived	11090	34·6	4009	25·1	2198	20·5	3550	27·8
Deprivation: England quintiles	Least Deprived	1975	50·1	1169	45·3	467	29·3	341	35·4
	Quintile 2	12521	45·8	5963	36·5	3633	29·8	2724	31·9
	Quintile 3	10910	42·2	4414	32·4	3261	28·4	2819	31·0
	Quintile 4	15928	41·3	7272	33·9	4067	26·8	3949	30·9
	Most Deprived	44172	37·2	17999	28·3	9405	23·2	12687	28·9

Table B3: Summary statistics for the numbers of positive tests. Note: Numbers <10 have been redacted due to statistical disclosure issues. Denominators for percentages are total number of tests per group.

Measure		6 Nov 2020 - 31 Jan 2021		6 Nov - 2 Dec 2020		3 Dec 2020 - 5 Jan 2021		6 Jan - 31 Jan 2021	
		**Frequency**	**Percentage**	**Frequency**	**Percentage**	**Frequency**	**Percentage**	**Frequency**	**Percentage**
Total tests		399603		166823		115242		117538	
Total positive tests		5192	1·30	827	0·50	1938	1·68	2427	2·06
Sex	Female	2604	1·18	434	0·47	1005	1·60	1165	1·77
	Male	2588	1·44	393	0·52	933	1·78	1262	2·44
Age band	6-14	297	0·92	100	0·45	81	1·49	116	2·55
	15-34	2309	1·65	315	0·64	961	2·06	1033	2·35
	35-69	2369	1·23	351	0·47	818	1·49	1200	1·90
	70+	217	0·63	61	0·30	78	0·93	78	1·30
Ethnic group	Asian	160	1·37	44	0·84	49	1·65	67	1·90
	Black	157	1·96	32	1·00	38	1·89	87	3·07
	Mixed	70	1·23	15	0·63	24	1·43	31	1·89
	Other	112	3·28	23	1·69	49	5·11	40	3·63
	White	4693	1·27	713	0·46	1778	1·65	2202	2·03
Deprivation: Liverpool quintiles	Least Deprived	1101	1·04	155	0·35	475	1·40	471	1·76
	Quintile 2	1053	1·07	169	0·41	443	1·49	441	1·59
	Quintile 3	1089	1·37	187	0·54	402	1·89	500	2·13
	Quintile 4	996	1·62	159	0·64	322	2·01	515	2·52
	Most Deprived	953	1·74	157	0·74	296	2·06	500	2·61
Deprivation: England quintiles	Least Deprived	57	0·71	<10	--	23	0·97	28	1·75
	Quintile 2	601	1·07	87	0·36	250	1·38	264	1·93
	Quintile 3	569	1·11	80	0·40	254	1·51	235	1·63
	Quintile 4	760	1·03	126	0·41	310	1·42	324	1·58
	Most Deprived	3205	1·52	528	0·60	1101	1·97	1576	2·34

Figure B1 presents the estimated percentage of the population in Liverpool who received a lateral flow test by 10 year age band. Uptake was lowest among the 6-9 and 80+ age groups, and highest among the 10-19 age group. The high uptake among individuals aged 10-19 mostly represents targeted testing among University students, as well as pilots targeting secondary schools and colleges.

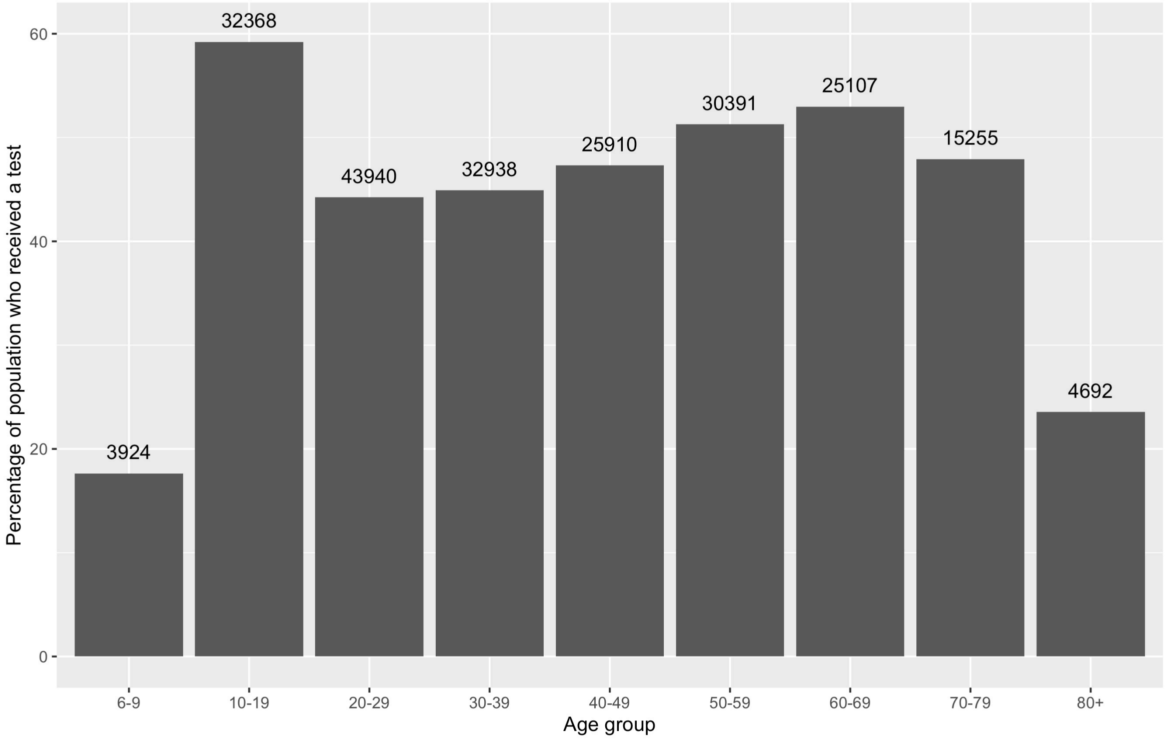

Figure B1: Percentage of people by ten-year age band who received a lateral flow test between 6th November 2020 and 13th Jan 2021 (frequency counts for uptake plotted above bars).

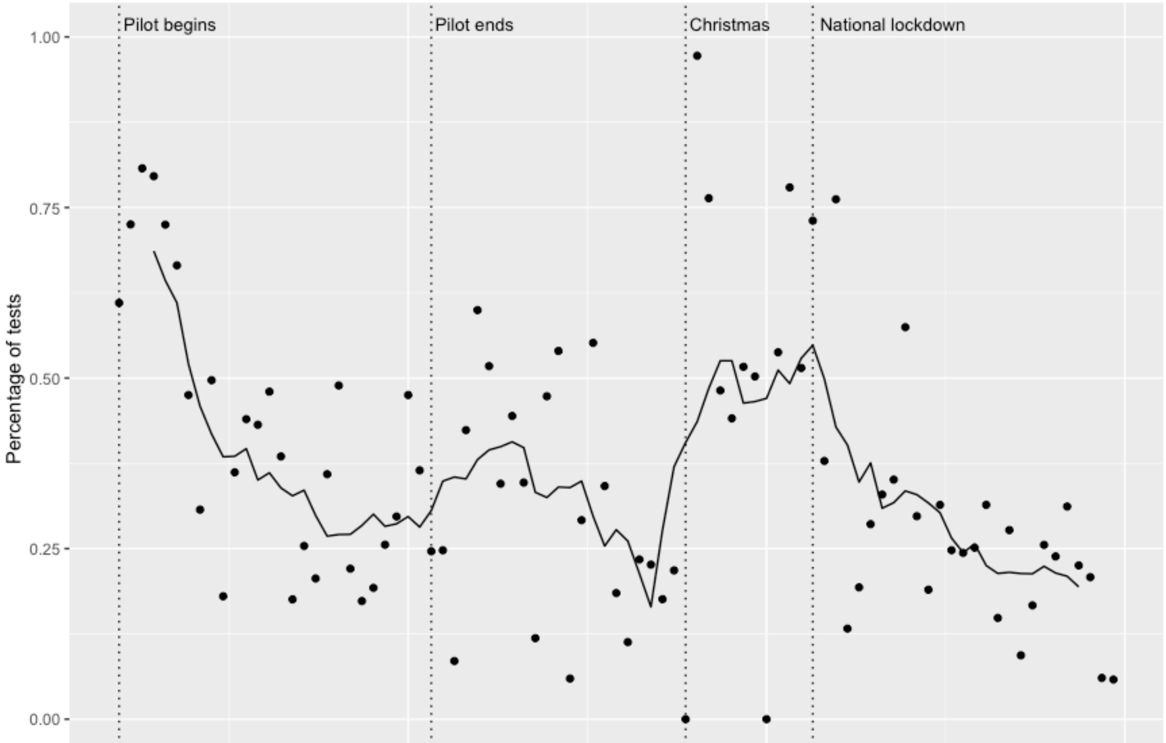

Figure B2: Percentage of people who received lateral flow tests who reported that they had symptoms. Note: points represent the raw daily value, with the line the 7 day average.

Figure B2 plots trends in the percentage of total lateral flow tests per day which were individuals who reported that they had symptoms of COVID-19 as they got a test. Overall prevalence was low (n = 1515, 0·38%). While the temporal trend remains low throughout the period, there are periods where the prevalence was higher including the start of the pilot and immediately following Christmas (the latter period reflecting a doubling of the previous week's values). Individuals could report whether they had any COVID-19 symptoms in the last 72 hours when registering their details prior to taking a lateral flow test. As such, these data are self-reported and likely to have under-estimated the numbers of symptomatic individuals who received lateral flow tests. If an individual showed up for a lateral flow test and said they had symptoms, the protocol was that they should have been redirected to a symptomatic test site, however this might have not always happened. We suggest caution in interpreting this variable, as we are unable to validate the quality and reliability of the measure.

Appendix C: Full analytical model results

The section presents full tables (Tables C1 to C3) of the spatial regression models that were presented in the main paper (Figures 2 to 4). Description of internet user classification area types can be seen in Appendix D.

Table C1: Relative risks (RR) for the associations between covariates and people having lateral flow tests per area. Note: Lower and upper limits refer to 95% Credible Intervals. Models adjusted for age, sex and ethnicity using indirect standardisation.

Variable	6th Nov - 13th Jan			6th Nov - 2nd Dec			3rd Dec - 5th Jan			6th Jan - 31st Jan		
	RR	lower	upper	RR	lower	upper	RR	lower	upper	RR	lower	upper
Deprivation score	0·86	0·80	0·91	0·81	0·74	0·89	0·82	0·78	0·87	0·87	0·83	0·92
Proportion students	0·96	0·91	1·01	0·95	0·89	1·03	0·93	0·89	0·97	0·91	0·87	0·94
Care home in area	1·15	1·07	1·24	1·18	1·06	1·31	1·03	0·97	1·10	1·11	1·04	1·18
Access to test site (km)	0·95	0·91	0·98	0·89	0·85	0·94	0·93	0·90	0·96	0·93	0·91	0·96
IUC (reference: e-Veterans)												
Digital Seniors	0·88	0·69	1·13	0·68	0·48	0·97	0·85	0·70	1·04	1·17	0·96	1·42
e-Cultural Creators	0·89	0·69	1·15	0·78	0·54	1·12	0·91	0·74	1·12	1·10	0·90	1·35
e-Mainstream	0·86	0·75	1·00	0·76	0·62	0·94	0·86	0·77	0·97	1·15	1·03	1·29
e-Professionals	0·92	0·77	1·10	0·83	0·64	1·07	0·91	0·79	1·06	1·01	0·87	1·16
e-Rational Utilitarians	0·93	0·73	1·19	0·83	0·59	1·18	0·98	0·81	1·20	1·06	0·88	1·29
e-Withdrawn	0·77	0·63	0·94	0·68	0·51	0·90	0·66	0·56	0·77	1·04	0·89	1·22
Passive and Uncommitted Users	0·82	0·70	0·96	0·74	0·59	0·94	0·72	0·63	0·82	1·04	0·92	1·18
Settled Offline Communities	0·89	0·60	1·32	0·70	0·40	1·23	1·09	0·79	1·50	1·38	1·01	1·89
Youthful Urban Fringe	0·79	0·63	0·98	0·72	0·53	0·99	0·76	0·63	0·90	0·95	0·80	1·13

Model fit (log marginal-likelihood) – Model for period 6th Nov – 13th Jan = -2015; Model for period 6th Nov – 2nd Dec = -1918; Model for period 3rd Dec – 5th Jan = -1664; Model for period 6th Jan – 31st Jan = -1644.

Table C2: Relative risks (RR) for the associations between covariates and people who received multiple lateral flow tests per area. Note: Lower and upper limits refer to 95% Credible Intervals. Models adjusted for age, sex and ethnicity using indirect standardisation.

Variable	6th Nov - 13th Jan			6th Nov - 2nd Dec			3rd Dec - 5th Jan			6th Jan - 31st Jan		
	RR	lower	upper	RR	lower	upper	RR	lower	upper	RR	lower	upper
Deprivation score	0·81	0·75	0·87	0·76	0·67	0·86	0·78	0·72	0·84	0·83	0·78	0·89
Proportion students	0·92	0·86	0·98	0·91	0·82	1·01	0·90	0·84	0·95	0·90	0·86	0·95
Care home in area	1·15	1·05	1·26	1·22	1·05	1·41	1·02	0·94	1·12	1·13	1·05	1·21
Access to test site	0·95	0·91	0·98	0·90	0·84	0·96	0·90	0·87	0·94	0·94	0·91	0·97
IUC (reference: e-Veterans)												
Digital Seniors	0·84	0·62	1·14	0·60	0·37	0·97	0·75	0·56	0·99	1·22	0·97	1·53
e-Cultural Creators	0·83	0·61	1·14	0·70	0·42	1·15	0·89	0·67	1·19	1·03	0·82	1·31
e-Mainstream	0·82	0·69	0·98	0·69	0·52	0·91	0·80	0·68	0·94	1·15	1·01	1·31
e-Professionals	0·86	0·69	1·08	0·75	0·53	1·06	0·92	0·75	1·13	0·96	0·81	1·14
e-Rational Utilitarians	0·91	0·67	1·23	0·79	0·49	1·27	0·98	0·75	1·29	1·07	0·85	1·35
e-Withdrawn	0·66	0·52	0·84	0·52	0·35	0·76	0·49	0·39	0·61	1·02	0·85	1·22
Passive and Uncommitted Users	0·72	0·59	0·88	0·61	0·44	0·84	0·60	0·50	0·72	1·03	0·89	1·20
Settled Offline Communities	0·86	0·53	1·40	0·67	0·31	1·46	1·16	0·75	1·80	1·43	0·99	2·05
Youthful Urban Fringe	0·73	0·55	0·95	0·62	0·40	0·96	0·71	0·55	0·91	0·93	0·75	1·14

Model fit (log marginal-likelihood) – Model for period 6th Nov – 13th Jan = -1787; Model for period 6th Nov – 2nd Dec = -1626; Model for period 3rd Dec – 5th Jan = -1341; Model for period 6th Jan – 31st Jan = -1330.

Table C3: Relative risks (RR) for the associations between covariates and positive lateral flow tests per area. Note: Lower and upper limits refer to 95% Credible Intervals. Models adjusted for age, sex and ethnicity using indirect standardisation.

Variable	6th Nov - 13th Jan			6th Nov - 2nd Dec			3rd Dec - 5th Jan			6th Jan - 31st Jan		
	RR	lower	upper	RR	lower	upper	RR	lower	upper	RR	lower	upper
Deprivation score	1·19	1·14	1·24	1·29	1·17	1·42	1·13	1·07	1·20	1·15	1·09	1·21
Proportion students	0·87	0·84	0·91	0·92	0·83	1·01	0·87	0·82	0·92	0·88	0·83	0·94
Care home in area	0·91	0·83	1·01	0·78	0·62	0·97	0·95	0·84	1·08	1·00	0·89	1·13

Model fit (log marginal-likelihood) – Model for period 6th Nov – 13th Jan = -938; Model for period 6th Nov – 2nd Dec = -598; Model for period 3rd Dec – 5th Jan = -709; Model for period 6th Jan – 31st Jan = -772.

Appendix D: Sensitivity analysis – alternative measures of internet usage

Our area level of digital inequality, internet user classification (Table 1), is useful since it is a multi-dimensional measure that combined information across a range of measures about confidence in using internet technologies. Key variables included in the construction of the classification of areas includes whether individuals own internet-enabled technologies, internet speed, how frequent individuals use the internet by different modes (e.g. laptop or mobile phone), information seeking behaviours online, use of social media or communication technologies, and online shopping habits. Descriptions of the area types are presented in Table D1.

Table D1: Description of Internet User Classification area types.

Area type	Description
e-Cultural Creators	High levels of internet use, especially for social media, communication, streaming and gaming. Typically younger and student populations.
e-Professionals	High levels of engagement and experienced users, especially for online shopping. Urban professionals and high educational attainment.
e-Veterans	Frequent use of internet technologies across multiple devises and uses. Typically more affluent suburbs.
Youthful Urban Fringe	Primarily mobile users or use in public places. Higher levels of communication. Young and ethnically diverse populations.
e-Rational Utilitarians	High demand, but with poor infrastructure (often due to rural areas). Low levels of mobile use, with higher personal computers. Often middle aged or older adults.
e-Mainstream	Average characteristics for most measures and reflect diverse populations
Passive and Uncommitted Users	Limited or no interactions with the internet. Often from suburbs or rural areas.
Digital Seniors	Average use of internet, often from personal computers. Mostly older adults.
Settled Offline Communities	Limited engagement of the internet, with poor access to infastructure. Online shopping when use services. Retired people in rural areas.
e-Withdrawn	Least engaged with the internet, with lowest access and use. Deprived populations.

Note: Descriptions taking from Singleton A, Alexiou A, Savani R. Mapping the geodemographics of digital inequality in Great Britain: An integration of machine learning into small area estimation. *Comput Environ Urban Syst* 2020;**82**:101486.

Internet User Classification is only a proxy measure of internet usage and its combination of variables may obscure specific associations. Here we re-run the analyses using a direct measure of data usage – median data usage (in gigabytes) for September 2019 produced by Ofcom (https://www.ofcom.org.uk/research-and-data/data/opendata). We suggest that these data are interpreted carefully, since median data usage in an area is not specifically confidence in using internet technologies (i.e. use is not necessarily the same as confidence, and skewed by streaming or downloads). Methods and data otherwise remain the same as presented in the main model. For brevity, we only present the results for the overall time period models (6th November 2020 to 31st January 2021). Continuous variables were centred and scaled to standardise them.

Table D2 presents summary statistics for each model. Standardised median data usage was negatively associated to both uptake (Relative Risk (RR) = 0·96, 95% Credible Intervals (CIs) = 0·92-0·99) and multiple tests (RR = 0·96, 95% CIs = 0·92- 1·00). The results suggest that in areas with greater internet usage, we observed fewer tests overall and fewer people who received multiple tests. Specifically, one standard deviation increase in the median data usage of an area would be associated with 4% fewer people having tests and 5% fewer people getting repeat tests. The finding is inconsistent with the result in the main analysis, and indeed our hypothesised directions. However, the credible intervals suggest some uncertainty in the associations.

Table D2: Relative risks (RR) for the associations between covariates for each outcome (6th November 2020 to 31st January 2021). Note: Lower and upper limits refer to 95% Credible Intervals. Bold results have credible intervals that do not contain 1.

	**Outcome: overall uptake (persons)**			**Outcome: multiple tests (persons)**		
	**RR**	**Lower**	**Upper**	**RR**	**Lower**	**Upper**
Deprivation score	0·82	0·79	0·85	0·74	0·71	0·77
Proportion of students in area	0·95	0·92	0·98	0·91	0·87	0·95
Care home beds / population	1·16	1·08	1·25	1·17	1·06	1·28
Average walking distance to nearest test site	0·95	0·92	0·98	0·95	0·92	0·99
Median data usage (GB)	0·96	0·92	0·99	0·96	0·92	1·00

Model fit (log marginal-likelihood) – Model for uptake = -1971; Model for multiple uptake = -1748.

We also tested whether there was an interaction effects between digital inequality and neighbourhood deprivation to assess whether the association between degree of deprivation and the outcome change with digital inequality. This was because these two issues are inter-related, with deprived communities often less able to afford internet related technologies. For example, the correlation between neighbourhood level deprivation score and median data usage in Liverpool was 0·47. Inclusion of interaction effects for models with both median data usage and internet user classification fit separately did not improve model fit, with interaction effects non-significant. The high correlation between deprivation and median data usage may partly explain the lack of association in the expected direction for median data usage, with the association reflecting the residual effect of deprivation. However, we also note that IUC is also correlated to deprivation.

Appendix E: Sensitivity analysis – individual level models

One limitation of ecological analyses is the ecological fallacy where inferences about relationships at the area level cannot be made for individuals. This is important where we are interested in how *people* respond to asymptomatic testing, rather than how *communities* respond. To extend the main analyses presented in the paper, we re-ran our models at the individual level. We present two models – one model for the likelihood of an individual having a positive test over the study period and one model for the likelihood of an individual having multiple tests over the study period. We were unable to undertake a similar analysis for lateral flow uptake as we did not have access to a full population register to identify who had received a test or not.

Multi-level binomial (binary) regression models were used to analyse each of our outcomes. The strength of the approach is the ability to independently test how individual level characteristics (level 1) and area level covariates (level 2; n = 298) were associated to our outcome. Models were run using the same area level covariates as presented in the main paper, with age, sex and ethnic group included as individual-level covariates. All continuous variables were standardised (centred and scaled using z-scores). Table E1 presents the model summary for both outcome measures. We divide the interpretation of the results by outcome measure. Relationships to likelihood of having had multiple (more than one) lateral flow test were often in the opposite direction compared to relationships to a positive test, suggesting that those populations less engaged with testing were also more likely to have benefitted from it.

Age was positively associated to likelihood of having multiple lateral flow tests, suggesting that older adults were more likely to have multiple tests. Males were less likely to have had multiple tests than compared to females. Each ethnic group was also less likely to have received multiple tests than compared to the 'White'

reference group. Deprivation was negatively associated to likelihood of having multiple tests, suggesting that individuals who lived in highly deprived areas were less likely to have had repeated testing. Individuals who lived in areas with student populations were less likely to have had multiple tests. There were no associations for care homes and access to test sites. Internet User Classification displayed several negative associations, suggesting that individuals who lived in areas characterised by communities less confident in using internet technologies were less likely to have received multiple tests.

For risk of having a positive test during the study period, age was negatively associated to the likelihood of a positive test. This would suggest that older adults were less likely to have tested positive. Males were more likely to have had a positive test compared to females. Few associations were detected by ethnic group, although individuals who were categorised in the 'Other' ethnic group were more likely to have had a positive test than compared to the 'White' reference group. In the area level covariates, deprivation was positively associated to likelihood of a positive test suggesting people who resided in deprived communities were more likely to have tested positive. Presence of a care home in an area was negatively associated to positivity, suggesting that individuals who lived in an area with a care home were less likely to have tested positive. Individuals who lived in areas with higher proportions of students were negatively associated to likelihood of a positive test.

Table E1: Summary results from multi-level binomial regression exploring the factors associated with individual level likelihood of a positive test or whether an individual had multiple tests (both for any point between 6th November 2020 to 31st January 2021). Note: CI = Confidence Interval. Estimates for random effects represent the variance and standard deviation.

	**Outcome: Multiple LFTs**			**Outcome: Positive test**		
	**Odds Ratio**	**Lower 95% CI**	**Upper 95% CI**	**Odds Ratio**	**Lower 95% CI**	**Upper 95% CI**
Individual level covariates						
Age	1·022	1.012	1·031	0·863	0·837	0·891
Sex:						
Female	Reference			Reference		
Male	0·847	0·832	0·862	1·127	1·063	1·194
Ethnic group:						
White	Reference			Reference		
Asian	0·682	0·647	0·719	0·946	0·801	1·117
Black	0·792	0·744	0·842	1·168	0·979	1·394
Mixed	0·924	0·859	0·994	0·866	0·670	1·118
Other	0·662	0·603	0·727	1·855	1·505	2·286
Area level covariates						
Deprivation	0·916	0·880	0·954	1·120	1·070	1·173
Proportion of students in area	0·933	0·899	0·968	0·846	0·799	0·895
Care home	0·990	0·944	1·038	0·874	0·784	0·974
Average distance to nearest test site	0·998	0·978	1·018			
Internet User Classification:						
e-Veterans	Reference					
Digital Seniors	0·795	0·681	0·928			
e-Cultural Creators	0·860	0·736	1·005			
e-Mainstream	0·906	0·830	0·990			
e-Professionals	0·880	0·788	0·982			

e-Rational Utilitarians	0·934	0·802	1·087		
e-Withdrawn	0·750	0·663	0·848		
Passive and Uncommitted Users	0·792	0·716	0·875		
Settled Offline Communities	0·938	0·734	1·199		
Youthful Urban Fringe	0·842	0·734	0·965		
Random effects (variance and standard deviation)					
Lower Super Output Area	0·023	0·153		0·091	0·302
Model fit					
AIC	282380			44663	
BIC	282596			44776	
Deviance	282338			44641	
Sample size (n)	214525			213009	

Appendix F – PCR adjudication of positive lateral flow tests

Individuals who received a positive lateral flow test (LFT) were asked to self-isolate and were expected to get a ‘confirmatory’ polymerase chain reaction (PCR) test to double-check the result. We assessed how commonly this occurred within our data. As the CIPHA resource utilised pillar 2 data (testing for the wider population) and did not include any pillar 1 records (testing in hospitals for individuals with clinical needs or among health or care workers), our findings may be underestimates. Table F1 presents the percentage of individuals who received a positive LFT and also got a follow up PCR test. During the initial pilot phase (6th November to 2nd December), follow up was poor, with 20% of individuals who received a positive LFT getting a PCR test within two days of their initial LFT. This was explained by the lack of clear messaging about the need to get a confirmatory PCR in the initial few weeks, where it was not recommended. The metric improved into the latter two periods, with almost half of all individuals who tested positive using a LFT also receiving a PCR on the same day. This reflects the improved messaging encouraging individuals to get a confirmatory PCR test.

Table F2 presents data for individuals who received a confirmatory PCR test, presenting the percentage of individuals who also received a positive result from their PCR test. Results that were void were excluded from the calculations. There was high agreement between the results for LFTs and PCRs overall (95% of positive LFTs with a confirmatory PCR within two days were also positive following a PCR test), although performance during the initial pilot was poorer (74%).

Table F1: Percentage of individuals who received a positive lateral flow test result that also received a follow up polymerase chain reaction (PCR) test.

Time for PCR test	6th November – 31st January	6th November – 2nd December	3rd December – 5th January	6th January – 31st January
Same day	47	15	51	49
Before end of next day	58	20	70	62

Table F2: Percentage of individuals who received a positive result for both a lateral flow test and polymerase chain reaction (PCR) test.

Time of PCR test	6th November – 31st January	6th November – 2nd December	3rd December – 5th January	6th January – 31st January
Same day	94	75	93	96
Before end of next day	93	74	93	95

We evaluated whether there were any inequalities in the likelihood of an individual having had a confirmatory PCR test. We used an individual level binomial multi-level model (individuals nested within LSOAs) to assess the likelihood of having a confirmatory PCR test or not (binary outcome). We focus on just individuals who had an immediate PCR test (i.e., before the end of the next day from when they had their positive LFT test). Individuals who had a confirmatory PCR within five days of a positive LFT test but after the next day were excluded from the analysis, since it was less likely such tests were part of the immediate follow up (inclusion these individuals do not significantly change the results of the analysis). We use the same covariates as used for assessing inequalities in uptake, other than Internet User Classification which was excluded since we did not hypothesise it would influence the likelihood of an individual getting a PCR test after being contacted to do so. Continuous variables were z-score standardised. Final sample size was 4726.

Table F3: Summary results from multi-level binomial regression exploring the factors associated with individual level likelihood of having had a confirmatory PCR test or not (6th November 2020 to 31st January 2021). Note: CI = Confidence Interval.

Variable	Odds Ratio	Lower 95% CI	Upper 95% CI
Individual level covariates			
Age	1·063	1·000	1·130
Sex:			
Female	Reference		
Male	0·985	0·874	1·110
Ethnic group:			
White	Reference		
Asian	0·412	0·289	0·588
Black	0·533	0·373	0·761
Mixed	0·754	0·451	1·260
Other	0·429	0·279	0·658
Area level covariates			
Deprivation	0·840	0·784	0·899
Proportion of students in area	0·843	0·788	0·901
Care home	0·952	0·815	1·113
Random effects (variance and standard deviation)			
Lower Super Output Area	0·055	0·234	
Model fit			
AIC	6242		
BIC	6313		
Deviance	6220		
Sample size (n)	4726		

Table F3 presents the model summary. Age was positively associated with likelihood of having had a confirmatory PCR either on the same day as a positive LFT test or the day after, suggesting that older adults were more likely to have received one. The estimated effect size was small though; converting the coefficient to the unstandardised values would suggest that for each one year increase in age, the odds of having had a confirmatory PCR test increased by 0.4% (Odds Ratio (OR) = 1.004, 95% Confidence Intervals (CIs) = 1.000-1.007). Individuals of Asian, Black or 'Other' ethnicity were less likely to have had a confirmatory PCR test than individuals of White ethnicity. Deprivation was negatively associated suggesting that as the area an individual lived in became more deprived, individuals were less likely to have had a confirmatory PCR test. The effect size could be interpreted as a one standard deviation increase in deprivation score (equivalent of going from Liverpool's third quintile to most deprived quintile) as associated to 16% reduction in the odds of having a confirmatory PCR test (OR = 0.840, 95% CIs = 0.784-0.899). Finally, our models suggests that individuals who lived in areas with higher student populations were less likely to have had a confirmatory PCR test.

We also repeated the analysis for likelihood of having had a positive test reported in both the LFT and PCR tests, however no associations were detected partly due to the high agreement (see Table F2) and we therefore do not report the models here.

Appendix G – Maps of each covariate

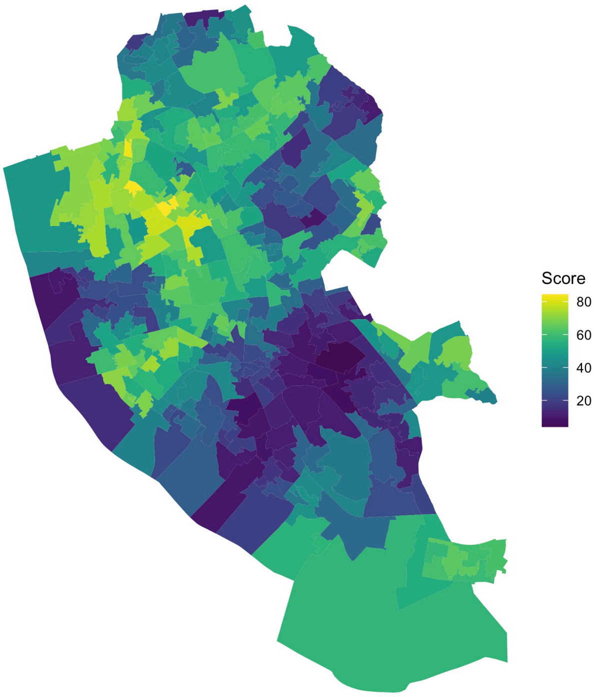

Figure G1: Geographical variation of index of multiple deprivation score (2019) for Liverpool.

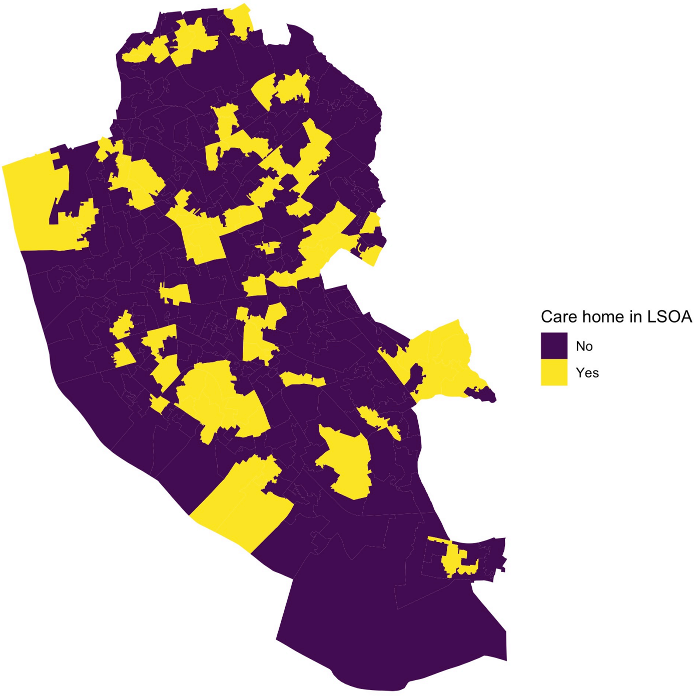

Figure G2: Location of care homes in Liverpool.

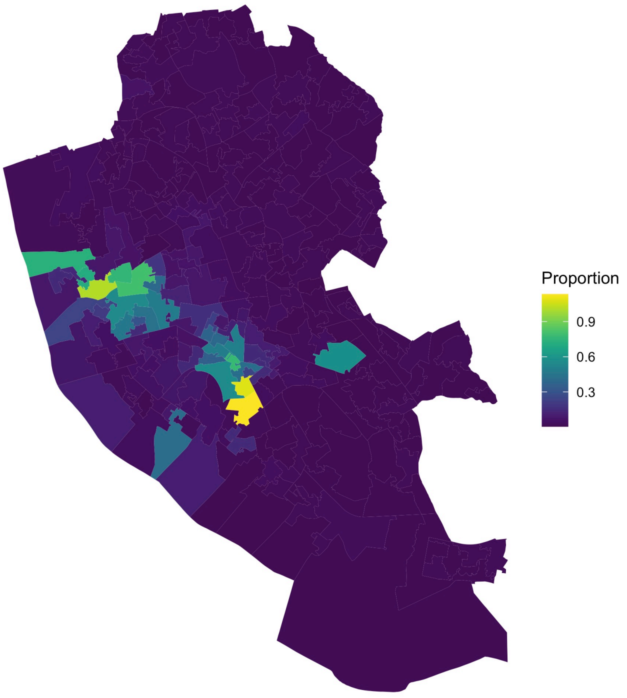

Figure G3: Proportion of residents in each lower super output area that were full time students (2011).

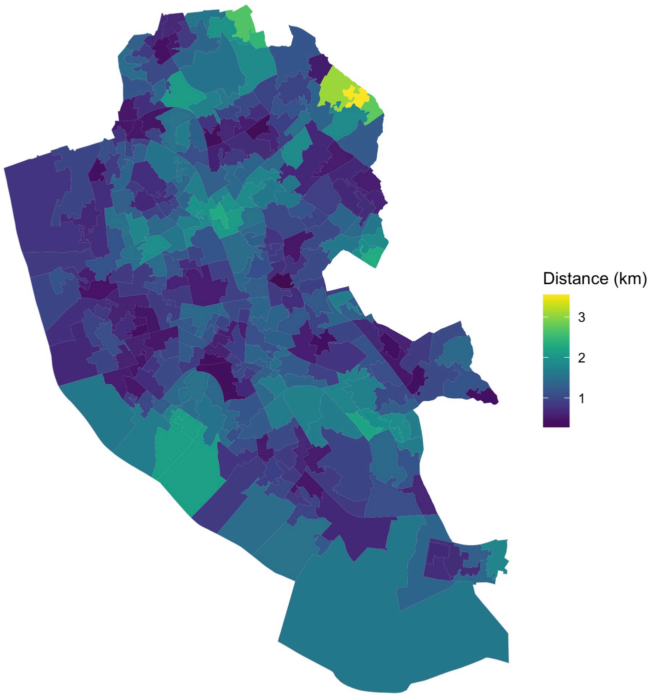

Figure G4: Mean walking distance to nearest asymptomatic test site during the main pilot period.

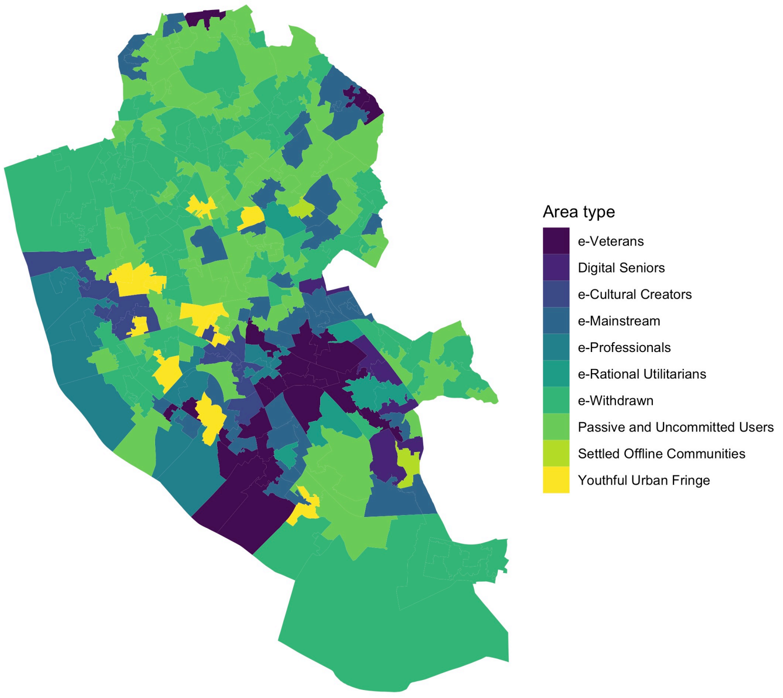

Figure G5: Location of Internet User Classification (2018) area types for Liverpool.
